# Genetic variation may confound analysis of CRISPR-Cas9 off-target mutations

**DOI:** 10.1038/s41421-018-0025-2

**Published:** 2018-04-17

**Authors:** Guanqun Wang, Meijie Du, Jianbin Wang, Ting F. Zhu

**Affiliations:** School of Life Sciences, Tsinghua-Peking Center for Life Sciences, Center for Synthetic and Systems Biology, Ministry of Education Key Laboratory of Bioinformatics, Tsinghua University, 100084 Beijing, China

Dear Editor,

The CRISPR-Cas9 system has been widely applied to animal genome editing^1–4^. Recently, Schaefer et al. identified the presence of >1300 single-nucleotide variants (SNVs) and >100 small insertions and deletions (indels) in CRISPR-edited mice using whole-genome sequencing (WGS)^5^. Nevertheless, no substantial homology between the SNV and indel regions with single guide RNA (sgRNA) sequences was found, and a large number of unique SNVs and indels were also present in the FVB/NJ control mouse^6, 7^, raising the question as to whether or not the majority of the observed mutations should be ascribed to the naturally occurring genetic variation of the inbred mice instead.

We happened to be in the process of generating a *Zkscan1* partial gene knockout (*Zkscan1*^+/-^) mouse strain using the CRISPR-Cas9 system (Materials and methods in Supplementary Information). To identify potential off-target mutations, we performed WGS and comparative analysis of the CRISPR-edited and control mice using standard DNA extraction, sequencing, and bioinformatic pipelines (Materials and methods in Supplementary Information). In light of the recently raised questions on the extent of genetic variation in the inbred animals and its potential confounding effect, we purposely sequenced three mice from the same production colony as controls.

We detected a total of 7416 SNVs and 1996 indels in all four mice (Fig. 1a), among which the CRISPR-edited mouse carried 725 unique SNVs and 57 unique indels. In comparison, the three control mice also harbored comparable amounts of unique SNVs and indels (Fig. 1a), and no significantly more variants were found in the CRISPR-edited mouse than in the controls (in fact, the highest number of unique SNVs was detected in one of the control mice). To further examine whether the CRISPR-edited animal genetically deviated from the control mice, we calculated the pair-wise discordance among the four mice by analyzing the number of SNV and indel sites at which the experimental mice have different genotypes compared with each other. We found that the pair-wise genetic discordance between the CRISPR-edited animal and each control was not higher than that between the control mice themselves (Fig. 1b; Supplementary Fig. S1 and S2). Because mutagenic processes most commonly result in heterozygous mutations, we analyzed the heterozygosity of the detected SNV and indel sites in four mice and found roughly equal levels of heterozygosity in the CRISPR-edited animal and the control mice (Supplementary Table S1).

**Fig. 1:**
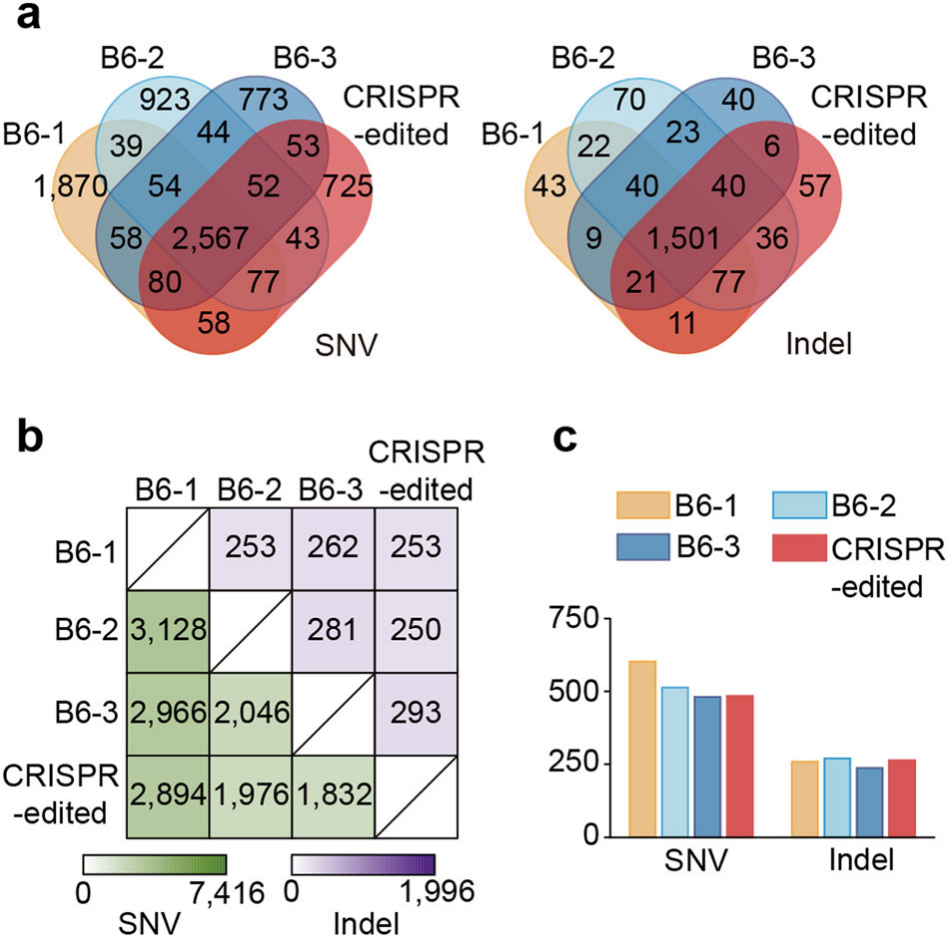
Genetic variants identified in the CRISPR-edited and control mice. **a** Venn diagrams displaying the number of SNVs (left) or indels (right) identified in the CRISPR-edited animal and control mice. B6-1, B6-2, and B6-3: C57BL/6J control mice. CRISPR-edited: *Zkscan1*^+/−^ mouse. **b** Heat maps showing the number of SNV (green) and indel (purple) sites at which the experimental mice have different genotypes compared with each other. **c** Number of SNV or indel sites located at 3-bp upstream from an NGG sequence (on both strands)

Prior studies on the CRISPR-Cas9 system indicated that *Streptococcus pyogenes* Cas9 (SpCas9)-mediated DNA cleavage requires the presence of an NGG protospacer adjacent motif sequence approximately 3-bp downstream from the cleavage site^8, 9^. We analyzed the flanking sequences of all the detected SNVs and indels in each experimental mouse to calculate the number of SNV or indel sites located at 1–5-bp upstream from an NGG sequence (Fig. 1c; Supplementary Fig. S3), and observed roughly equal amounts of such sites in the CRISPR-edited animal and the control mice. We also analyzed the potential off-target sites bioinformatically by the CRISPR Design tool^10^ for the two sgRNA targets (Supplementary Table S2), with 71 potential off-target sites for sgRNA-1 and 15 for sgRNA-2 predicted (Supplementary Table S3); however, no overlaps between the potential off-target sites and SNV or indel regions were found.

The extent of off-target mutations in a CRISPR-edited animal are undoubtedly influenced by various factors such as sgRNA design and the specific genome-editing protocol used, and more studies are needed to comprehensively understand CRISPR-Cas9 off-targeting. Based on our results, we did not find enough evidence to ascribe the majority of the SNVs and indels detected in this study to the off-target effects of CRISPR-Cas9 editing per se. Instead, we found that the extensive genetic variation in inbred experimental animals could become a significant confounding factor in the analysis of CRISPR-Cas9 off-target mutations, which needs to be properly assessed by well-designed control experiments in future studies.

While this work was being peer reviewed, another article was uploaded to preprint server, reporting the trio sequencing of CRISPR-edited mice and pedigree-matched controls^11^, the main findings of which are consistent with those in our work.

## Data availability

Sequencing data are available at SRA: BioProject PRJNA419684 (accession SRP126009). A UCSC Genome Browser Track Hub (https://genome.ucsc.edu/cgi-bin/hgHubConnect) is available for import from URL: https://de.cyverse.org/dl/d/27589272-8544-466E-8242-418BB8B9BED3/hub.txt.

## Electronic supplementary material

Supplementary Information

Dataset 1

Dataset 2
