# Supplementary material for: Genetic variation may confound analysis of CRISPR-Cas9 off-target mutations
Source: Cell Discov. 2018 Apr 17;4:18. doi: 10.1038/s41421-018-0025-2 (PMC5902475; doi:10.1038/s41421-018-0025-2)
Supplement: Supplementary file 1 — Supplementary Information [file 41421_2018_25_MOESM1_ESM.pdf]

Supplementary Information

Genetic variation may confound analysis of CRISPR-Cas9 off-target mutations

Guanqun Wang[1,2], Meijie Du[1,2], Jianbin Wang[1]*, Ting F. Zhu[1]*

[1]School of Life Sciences, Tsinghua-Peking Center for Life Sciences, Center for Synthetic and Systems Biology, Ministry of Education Key Laboratory of Bioinformatics, Tsinghua University, Beijing 100084, China

[2]These authors contributed equally to this work.

*Correspondence: J.W. (jianbinwang@tsinghua.edu.cn) or T.F.Z. (tzhu@tsinghua.edu.cn).

Materials and Methods

CRISPR-edited mouse

All animal studies were approved by the Institutional Animal Care and Use Committee of Tsinghua University (Protocol: 17-ZT-1). All C57BL/6J mice were purchased from Vital River Laboratory Animal Technology (Beijing, China). *Zkscan1*$^{+/-}$ mice were constructed as previous described[1] with the following modifications: (1) sgRNA sequences were designed online (http://zifit.partners.org/ZiFiT/CSquare9Nuclease.aspx) and target specificity of sgRNA in mice was screened by Ensembl Blast; (2) Cas9 mRNA were *in vitro* transcribed using mMESSAGE mMACHINE SP6 Kit (Ambion, USA) from pSP6-2sNLS-spCas9 plasmid[2] linearized by XbaI (New England BioLabs, USA), and poly (A) tailing of Cas9 mRNA was conducted with *E. coli* Poly(A) Polymerase (New England BioLabs, USA) to increase Cas9 mRNA stability; (3) sgRNAs were *in vitro* transcribed by pT7-gRNA expression plasmid[2] using MAXIscript T7 Transcription Kit (Ambion, USA) and purified by mirVana miRNA Isolation Kit (Ambion, USA); (4) 200 zygotes obtained by mating males with superovulated C57BL/6J females were cytoplasmic injected with a mixture of purified Cas9 mRNA (80 ng/μl) and sgRNAs (40 ng/μl), and the injected zygotes were transferred into pseudo-pregnant C57BL/6J female mice, where the offsprings were obtained; (5) Effective gene knockout in offspring was determined by PCR on genomic DNA extracted from animal tail. The *Zkscan1* partial gene knockout was confirmed by whole genome sequencing.

Genomic DNA extraction, library preparation, and whole genome sequencing

Three C57BL/6J mice from the same production colony were used as controls (B6-1: 6-month-old

C57BL/6J control mouse; B6-2 and B6-3: 1-month-old C57BL/6J control mice; CRISPR-edited: 8-month-old *Zkscan1*$^{+/-}$ mouse). Total genomic DNA was extracted from 0.5 cm tails from the CRISPR-edited *Zkscan1*$^{+/-}$ mouse and three control mice using the HiPure Tissue DNA Mini Kit from Magen (Guangzhou, China). Notably, we used mechanical disruption in liquid nitrogen instead of overnight enzymatic digestion to lyse the tissue samples in order to minimize damage to the extracted genomic DNA. Genomic DNA quality was determined by Qubit (Thermo Fisher Scientific, USA) and Agilent 2100 Bioanalyzer (Agilent, USA), and sequencing libraries were prepared by the NEBNext Ultra II DNA Library Prep Kit for Illumina (New England BioLabs, USA). Libraries were sequenced on an Illumina HiSeq X10 sequencer using 2×150 bp cycles to an average depth of 25×.

SNV and indel calling

The Illumina reads were aligned to Mouse Genome Assembly GRCm38 by Bowtie2 (filter bam with MAPQ≥30, and AS>-10). To ensure unique mapping, we filtered the bam file by removing all lines with the XS (alternative mapping score) tag. We retained only positions with sequencing depth ≥10× in all experimental mice. SNVs and indels were called by GATK (GenomeAnalysisTK-3.5) and Pindel (pindel-0.2.0), respectively. SNVs were filtered with dbSNP142. SNVs and indels within or adjacent to a homopolymer stretch ≥5 bp were excluded from downstream analyses. Heterozygosity analysis was performed using an alternative allele frequency cutoff between 30-70% and depth ≥5×; homozygosity analysis was performed using an alternative allele frequency cutoff >70%.

Prediction of off-target sites

The potential off-target sites were predicted using the CRISPR Design tool (http://crispr.mit.edu/)[3],

with genome coordinates converted from mm9 to mm10 online (http://genome.ucsc.edu/cgi-bin/hgLiftOver).

Supplementary References

1 Wang, H. *et al.* One-step generation of mice carrying mutations in multiple genes by CRISPR/Cas-mediated genome engineering. *Cell* **153**, 910-918 (2013).

2 Wei, W. *et al.* Heritable genome editing with CRISPR/Cas9 in the silkworm, Bombyx mori. *PLoS One* **9**, e101210 (2014).

3 Hsu, P. D. *et al.* DNA targeting specificity of RNA-guided Cas9 nucleases. *Nat Biotechnol* **31**, 827-832 (2013).

Supplementary Figures

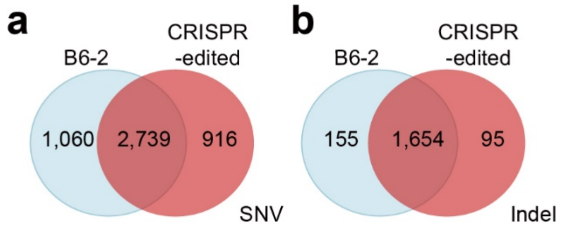

Figure S1. Venn diagrams displaying the number of SNVs **(a)** and indels **(b)** at which the B6-2 and CRISPR-edited mice have different (Fig. 1b) or same genotypes (Figure S2).

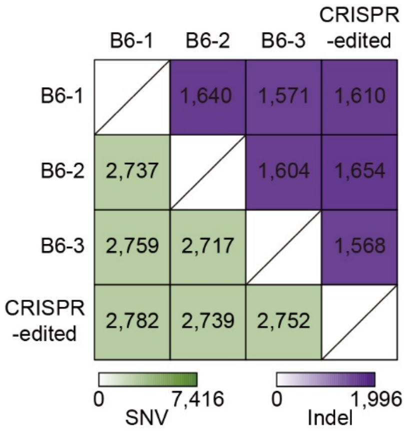

Figure S2. Heat maps showing the number of SNV (green) and indel (purple) sites at which the experimental mice have different genotypes from the reference genome but same compared with each other.

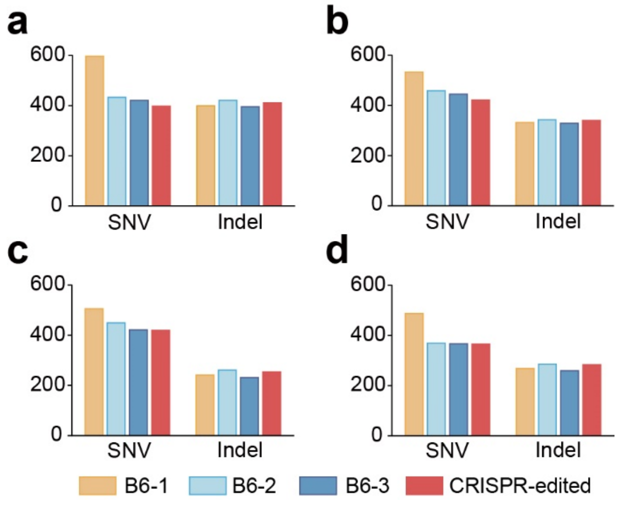

Figure S3. Number of SNV or indel sites located at 1 bp **(a)**, 2 bp **(b)**, 4 bp **(c)**, or 5 bp **(d)** upstream from an NGG sequence (on both strands).

Supplementary Tables

Table S1. Heterozygous SNVs and indels

Type	Mouse	Not excluding homopolymers ≥5 bp	Excluding homopolymers ≥5 bp
**SNV**	B6-1	2933	2765
	B6-2	1739	1634
	B6-3	1679	1569
	CRISPR-edited	1700	1592
**Indel**	B6-1	1339	332
	B6-2	1490	318
	B6-3	1156	283
	CRISPR-edited	1297	309

Table S2. sgRNA-targeted sequences

sgRNA-1 target	CTTTCCACCATGCCGGTCGC(AGG)
sgRNA-2 target	(CCA)TGTACTCGCCGAACATTACG

Table S3. Predicted off-target sites

sgRNA-1:

chr	strand	position	off-target sequence	mismatches	score
chr5	-	118312761	ATTTCCACCATGCCGGCCACCGG	3	0.433941
chr5	+	128703328	CATTCCCTCATGCCGGTCACGGG	4	0.353205
chr11	+	46127325	CTCTCCAACATGCCGGACACTAG	4	0.192757
chr4	+	154418388	CTTACCTCCTTGCCGGTGGCTGG	4	0.191803
chr1	-	93376857	CATACCACCAGGCCGGTGGCAAG	4	0.175352
chr13	-	83579732	CTTTCCAACCTCCCTGTCGCAAG	4	0.168338
chr7	-	112878966	CTTTCTACTCTGCCGGTCTCAGG	4	0.16397
chr1	+	23102221	CTGTCCGCCATGGCGGCCGCTGG	4	0.156095
chr5	+	99128629	CTTTCCAGCATGCCTGTTGCCAG	3	0.147857
chr3	+	51252483	CTTCCCAACATGCCAGTCCCCAG	4	0.133665
chr4	+	57297553	CTTCCCAACATGCCAGTCCCTGG	4	0.133665
chr3	+	89386119	CTCTCCACCGTGCCTGTCCCTGG	4	0.123579
chr11	+	75769779	CTTCCCACCCTGCCTGTCCCTGG	4	0.123105
chr14	-	56131915	CTCTCCACTCTGCAGGTCGCCAG	4	0.122209
chr11	-	84871069	CTTTCCACCCCACCGGTCTCCAG	4	0.11334
chr4	+	118402816	CTTACCACAATGCCAGTCGATGG	4	0.110071
chr7	+	128419844	CTTCCCACCGTGCCGGCAGCAAG	4	0.108117
chr2	-	156445212	CTGTCCACCAGGCCGGCCACGGG	4	0.10698
chr1	+	159449750	CTTTACAGCATGCTGGTCGATAG	4	0.098377
chr17	-	12159397	CTTTCCAACATGGCGGACGGGAG	4	0.093393
chr11	-	80996065	CTTGTCACCATGCCGCTCCCCAG	4	0.085785
chr1	-	40064679	CTTCCCACGATGCCTGTCTCTGG	4	0.081669
chr9	-	23534836	TTTTCCATCATGCTGGTCCCTGG	4	0.0785
chr7	-	28820174	ATTTCCACCATGGCGGCCCCCGG	4	0.078498
chr2	+	58911126	CTTTCAAACATGCCGGTGGTGGG	4	0.076925
chr12	+	60496636	CTTTCCATGATGCCTGTCACAAG	4	0.076247
chr14	+	69122353	CTCTCCATCATGCAGGTCTCAAG	4	0.0746
chr2	+	178536233	CTTTCCCCAATGTCTGTCGCCAG	4	0.060946
chr12	-	98577328	CCTTCCCCCATGCCAGTGGCCAG	4	0.057833
chr5	+	105418777	CTCTCCACCATGCCTGTCCTTAG	4	0.056984
chr8	-	111069746	CCTTCCACAATGCTGGACGCTGG	4	0.055496
chr19	+	6220447	GTTTCCACCATGCCTGGCTCTAG	4	0.05436
chr2	+	26906573	CTTTCTCCCATGCCTGTCCCCAG	4	0.053336
chr6	-	136172101	CTTTCATCCATGCCTGTCCCAGG	4	0.053336
chr3	+	106788186	ATTTCTACCATGCCTGTGGCAGG	4	0.052172
chr12	+	106178507	CTCTCCTCCATGCTGGTCCCTGG	4	0.050952
chr4	+	45912550	CTCTCCACAATGCCAGTTGCCAG	4	0.050105

chr12	+	88736181	CTTTCCTCCCTGCCTGTTGCTAG	4	0.048844
chr5	+	116553226	CTTTCCAGCATGTCTGTCCCTGG	4	0.048294
chr12	+	3906985	CTTTCCAGCATGCCAGGCCCTGG	4	0.048045
chr7	-	104083610	TTTTCCAACATGCAGGTGGCCAG	4	0.047944
chr1	+	169043676	CTTTCCACCATGAGGGTCCCAGG	3	0.0462
chr7	+	115521671	CTGTTCACCATGCAGGTGGCAAG	4	0.045592
chr5	+	52327615	CTTTCCACCTTGACAGTCCCCGG	4	0.04305
chr13	-	4038091	CTTCCCACCATTCTGGTCACAAG	4	0.036562
chr5	-	122208940	CTGTCCTCCATGCTGGTGGCAAG	4	0.03114
chr2	+	44947693	CTCTCCACCATGCCCTTCGTCAG	4	0.031115
chr15	+	82849186	ATTTCCACCATGTAGGTCCCAGG	4	0.03038
chr10	-	91614389	CTTTCCAACATGTCTGTTGCTAG	4	0.029559
chr16	+	77182800	CTTTCCAACATGCCTGGAGCCAG	4	0.029406
chr17	+	47939651	CTTTCCACAATGTCAGTCCCAGG	4	0.029026
chr5	-	5341981	CTTACAACCATGCAGGTGGCTAG	4	0.027486
chr2	-	152486198	CTTTCCTCCCTGCAGGTGGCTGG	4	0.027156
chr4	-	43636649	CTTTCCTCCATTCCCGTGGCAAG	4	0.026092
chr8	+	26485735	CTTTCCACTAAGCCAGTGGCCAG	4	0.025485
chrX	-	160217031	CATTCCACCATACTGGTGGCCAG	4	0.023162
chr2	-	73374734	CTTTCCTCCATGCCAGGTGCCAG	4	0.020418
chr9	+	3160679	CTTTCCAGCATGCCGTCTGCCAG	4	0.018873
chr1	+	133209922	CTTTCTCCCATGCTGGTTGCTGG	4	0.018139
chr14	-	67857769	CTTCCCACCATGCTGGAAGCCAG	4	0.017491
chr12	-	56720772	CTTTCCATCATGCTGGGGGCAAG	4	0.016349
chrX	-	36423467	CTTTCAACCAAGCAGGTGGCAAG	4	0.01474
chr11	+	7764185	CTTTCTACCATGCAGGTGGGTGG	4	0.011462
chr14	+	55004761	CTTTCCCCCATGCCCTTGGCTAG	4	0.009122
chr11	-	82837831	ATTTCCACCATGCTGCTGGCCAG	4	0.008246
chr4	-	97774035	GTTTCCACCATGCAGATGGCCAG	4	0.008246
chr17	+	23922982	CTTTCCACTATGCAGTTCCCAGG	4	0.007172
chr15	-	52920375	CTTTCCACCATGCATGTCAAAAG	4	0.007159
chr19	+	40979078	CTTTCCACCATGTAAGTCCCAGG	4	0.006644
chr10	-	75642778	CTTTCCACCATGCCACTTGGTAG	4	0.005065
chr12	-	98570232	CTTTCCACCATGCGACTCTCAGG	4	0.002908

sgRNA-2:

chr	strand	position	off-target sequence	mismatches	score
chr7	+	73538015	CCTGCTGTTTGGCGAGTACACAG	4	1.296863
chr6	-	55804114	GGCAATGTCTGGCGAGTACAGGG	4	0.793841
chr16	+	75695967	CGTTCTGCTCGGGGAGTACATAG	4	0.55369

chrX	+	132512483	CATGATGTTGGGTGAGTACAGAG	4	0.526876
chr4	+	117669821	CTTTAAGTTCTGCGAGTACAGGG	4	0.480401
chr12	-	33574002	TGTAATGATCAGAGAGTACAAAG	4	0.322857
chrX	+	109559260	GGTAATGATCTGAGAGTACACAG	4	0.322857
chr6	-	6843850	TGGAATGTTCAGAGAGTACAAGG	4	0.318337
chr1	+	14519407	TGAAATGTTGGGCGAGTTCAGAG	4	0.292205
chr11	-	117993557	CGGAATGTCCAGAGAGTACAGAG	4	0.188153
chr6	-	12443614	CCTAATTTTCGGAGAGAACATAG	4	0.161126
chr10	+	70878691	CCTAATGATCGGTGTGTACATAG	4	0.158579
chr16	-	59475747	GGTAATATTTGGCCAGTACAGGG	4	0.143306
chr6	-	124303958	CGTAATGTATGGGTAGTACATAG	4	0.043622
chr7	-	104635348	CGTAATGTTCAGTGTGTAAAAAG	4	0.025532

Supplementary Data

Dataset 1. Identified SNVs in four experimental mice (not excluding homopolymers ≥5 bp)

Dataset 2. Identified indels in four experimental mice (not excluding homopolymers ≥5 bp)
